# Inactivation of miR-100 combined with arsenic treatment enhances the malignant transformation of BEAS-2B cells via stimulating epithelial -mesenchymal transition

**DOI:** 10.1080/15384047.2017.1345393

**Published:** 2017-11-20

**Authors:** Jia Yang, Zhijun Chen, Xinyi Wang, Mo Xu, Haoshu Fang, Feifei Li, Yakun Liu, Yu Jiang, Yi Ding, Juan Li, Siying Wang

**Affiliations:** a Department of Anesthesia, School of Medicine, Shandong University, Jinan, Shandong, China; b Department of Pathophysiology, School of Basic Medical Science, Affiliated Anhui Provincial Hospital, Anhui Medical University, Hefei, Anhui, China; c Department of Pathology and Physiology, Weifang Medical College, Weifang, Shandong, China

**Keywords:** Carcinogenesis, lung cancer, micro RNA, miR-100

## Abstract

Chronic arsenic treatment induces epithelial-mesenchymal transition (EMT) and promotes tumorigenicity, but the mechanism is unclear. MiR-100 has been shown to be involved in this biologic process. In this study, we hypothesize that inactivation of miR-100 combined with low concentration of arsenic exposure could promote the malignant transformation of human bronchial epithelial cells (BEAS-2B cell) by promoting EMT. To test this hypothesis, BEAS–2B cells were treated with low-dose of As_2_O_3_ chronically, and lentiviral vectors were used to mediate the inhibition of miR-100 expression. Flow cytometry, cloning formation, and transwell assays were used to examine cell cycle progression, cell proliferation, and cell migration, respectively. The mouse xenograft model was used to investigate the cell malignant growth in vivo, and western blot was used to detect EMT related marker expressions. Our results showed that, the inactivation of miR-100 combined with arsenic treatment significantly promoted the proliferation, viability, and migration of BEAS-2B cells in vitro, and tumorigenesis in vivo. Consistently, the EMT related marker expressions were also significantly increased in corresponding groups. Our data indicate that inactivation of miR-100 combined with chronic arsenic treatment promotes tumorigenicity of BEAS-2B cells via activation of EMT. This novel insight may help us to better understand the pathogenesis of arsenic carcinogenesis.

## Introduction

Lung cancer is the leading cause of mortality worldwide.^1^ The occurrence of lung cancer is most commonly associated with the air and water pollution. Arsenic is a toxic heavy metal existing as a mixture in the atmospheric environment and water, and considered as a risk factor of lung cancer. Chronic arsenic exposure from contaminated drinking water and air has been reported in many countries.^2^ Study indicated that human bronchial epithelial cells (BEAS-2B) cells that were chronically exposed to sodium arsenite increase proliferation and a certain degree of malignant transformation.^3^ Although the carcinogenic evidence of arsenic in humans has been widely observed, the mechanisms are still unclear.

The tumorigenesis is a long-term process, which is influenced by both environmental and genetic factors in multi-factorial fashion.^4-6^ The abnormal expression of miRNAs might promote the carcinogenesis of lung cancer.^7^ The research about the relationship between miR-100 and tumor has made significant progresses, but the data so far are still controversial.^8^ Study found that, in prostate cancer, the miR-100 expression was elevated and associated with increased metastasis.^9^ However, in lung cancers, the expression of miR-100 was downregulated, suggesting it played a tumor suppressor function.^10-13^

Epithelial-mesenchymal transition (EMT) is regulated by transcription factors^14^^,^^15^ extracellular ligands and microRNAs.^16-18^ It has been proposed that inducing EMT in epithelial tumor cells enhances migration, invasion and dissemination, whereas the MET process facilitates metastatic colonization.^14^^,^^15^^,^^19^ In addition, induction of EMT in differentiated tumor cells has been shown to generate cells with properties of tumor-initiating cells, or cancer stem cells.^20^

In present study, both in vitro and in vivo experiments were performed to test our hypothesis that downregulation of miR-100 combined with chronic arsenic exposure could enhance metastasis and proliferation of BEAS-2B by promoting EMT, and our results confirmed this notion.

## Materials and methods

### Cell culture and reagents

The BEAS-2B cell line was obtained from the American Type Culture Collection. Cells were maintained in 5% CO_2_ at 37°C in Dulbecco's modified Eagle's medium (DMEM), supplemented with 10% fetal bovine serum(FBS, Life Technologies/Gibco), 100 U/mL penicillin, and 100 ug/mL streptomycin (Life Technologies/Gibco). Cell culture flasks used should be pre-coated with a mixture of 0.01mg/ml fibronectin, 0.03 mg/ml bovine collagen type I and 0.01 mg/mL bovine serum albumin dissolved in DMEM. For arsenic chronic treatment, 1 × 10^5^ cells were seeded into 6-cm dishes for 12 h and maintained in 0.25 μM As_2_O_3_ (Sigma) for 48-72 h per passage. This process was continued for about 10 weeks (20 passages) and 20 weeks (40 passages). For arsenic acute stimulate, 5 μM As_2_O_3_ (Sigma) was co-cultured with BEAS-2B cells with or without miR-100 inhibition for 0 h, 6 h, 12 h, and 24 h, respectively.

### Lentivirus-mediated suppression of miR-100–3p

The lentivirus was obtained from Genechem (Shanghai, China). For control or miR-100–3p inhibition group, a sequence encoding a miR-100–3p negative control or its specific inhibitor was cloned into the lentiviral vector hU6-MCS-Ubiquitin–EGFP -IRES-puromycin. BEAS-2B cells (1 × 10^6^) were infected with 1 × 10^7^ lentivirus transducing units in the presence of 10 μg/ml polybrene (Sigma-Aldrich).

### Methyl Thiazolyl Tetrazolium (MTT) assay

Arsenic treated BEAS-2B (miR-100-inhibitor) and BEAS-2B (miR-NC) cells were seeded and cultured on 96-well plates at an initial density of 2000/well after trypsinization. The cell's viability was measured by assay at 0, 24, 48, 72, and 96 hours. Specifically, 0.02 mL of MTT solution (5 mg/ml in PBS) was added into each well, and incubated for 4 hours at 37°C. After that, the medium was replaced by 0.15 mL of dimethyl sulfoxide for 15 min incubation. The optical density at 490 nm was measured by 96 well-plate spectrophotometer (Thermo Scientific, MA). All experiments were performed in triplicate.

### Cell cycle analysis

Arsenic treated BEAS-2B (miR-100-inhibitor) and BEAS-2B(miR-NC) cells were harvested. 1 × 10^6^ cells collected after washing twice with PBS, and fixing in cold ethanol (70%) for overnight. After washing with PBS, cells were permeabilized with 100 μL RNAase in PBS for 30 min at 37°C in the absence of light, and then cells were stained with 500 μL of propidium iodide (PI) for 30 min. The cell-cycle phases were analyzed by flow cytometry system (BD Biosciences, Bedford, MD, USA) at an excitation wavelength of 488 nm and an emission wavelength of 525 nm.

### Colony-formation assay

Arsenic treated BEAS-2B (miR-100-inhibitor) and BEAS-2B(miR-NC) cells were seeded and cultured on 60 mm^2^ plates at an initial density of 400/well after trypsinization, each group was measured in 3 parallel wells, and incubated for 2 weeks at 37°C, 5% CO_2_. Then cells were washed with PBS, and fixed for 15 minutes at room temperature. Cells were then stained with crystal violet 10∼30 minutes, washed and air-dried. Colony numbers were counted.

### Soft agar assay for colony formation

One thousand arsenic treated BEAS-2B (miR-100-inhibitor) and BEAS-2B (miR-NC) cells were re-suspended in 1mL of complete medium (DMEM medium with 10%FBS) containing 0.6% agar and were then plated on top of a bottom layer that contains 1.2% agar (BD) with complete medium. Plates were cultureed under normal conditions for 2 weeks, and then cells colony were counted and photographed under a microscope (Nikon).

### Cell migration assays

For the migration assays, a total of 2 × 10^4^ arsenic treated BEAS-2B(miR-100 -inhibitor) and BEAS-2B (miR-NC) cells in serum-free media were placed into the upper chamber of an insert (8 μm pore size, millepore). The chambers were then inserted into a 24 well culture plate and filled with DMEM medium containing 10% FBS. After 8 h, the cells remaining on the upper surface of the membranes were scraped off, whereas, the cells located on the lower surface were fixed, stained with 0.1% crystal violet, imaged, and counted under a microscope (Olympus, Tokyo, Japan). The same experiments were independently repeated 3 times.

### Western blotting assays

Western blotting was performed according to the standard procedure. Briefly, cultured cells were lysed in RIPA buffer supplemented with complete protease inhibitor (Roche, Mannheim, Germany). Aliquots (60∼80 μg) of total protein extracts were resolved on 6∼10% sodium dodecyl sulfate PAGE (SDS-PAGE) gels and transferred to polyvinylidene fluoride membranes (PVDF, Millipore). The membranes were then incubated with antibodies against E-cadherin (1:1000; Cell Signaling Technology, USA), Vimentin (1:1000; Cell Signaling Technology, USA), ZEB-1 (1:1000; Cell Signaling Technology), MMP-3 (1:1000; Cell Signaling Technology, USA), MMP-9 (1:1000; Abcam, USA), β-catenin (1:2000; Cell Signaling Technology), β-actin (1:10000; Santa Cruz Biotechnology, Santa Cruz, CA). Subsequently, the membranes were incubated with specific HRP-conjugated secondary antibodies (1:5000; Sigma-Aldrich, St Louis, MO, USA). Signals were detected using the enhanced chemiluminescence western blotting system (ComWin Biotech, Beijing, China).

### In vivo tumorigenicity assay

Tumor cells (5 × 10^5^) were diluted with PBS to a total volume of 0.1 ml. The tumor cells were subcutaneously inoculated into 6-week-old male BALB/C nude mice (5 mice per group) on the flank regions of legs of both sides. The length and width of the tumors were monitored every 5 d. The tumor size was calculated by the formula: width × length × (width + length)/2. After 90 d of observation, the mice were sacrificed and the solid tumor was harvested. All animal experiments were approved by the Institute Research Ethics Committee of Anhui Medical University.

### Statistical analysis

The data are expressed as the mean ± SD of at least 3 independent experiments. The x^2^ test or rank test was used for categorical variables, and Student's T-test for continuous variables. The sigma plot version 12.5 (systat-software) was used for statistical analysis and figure creation. *indicates statistical difference with p < 0.05, **indicates statistical difference with p < 0.01.

## Results

### Inactivation of miR-100 combined with arsenic promotes proliferation of BEAS-2B cells

**Figure 1.**
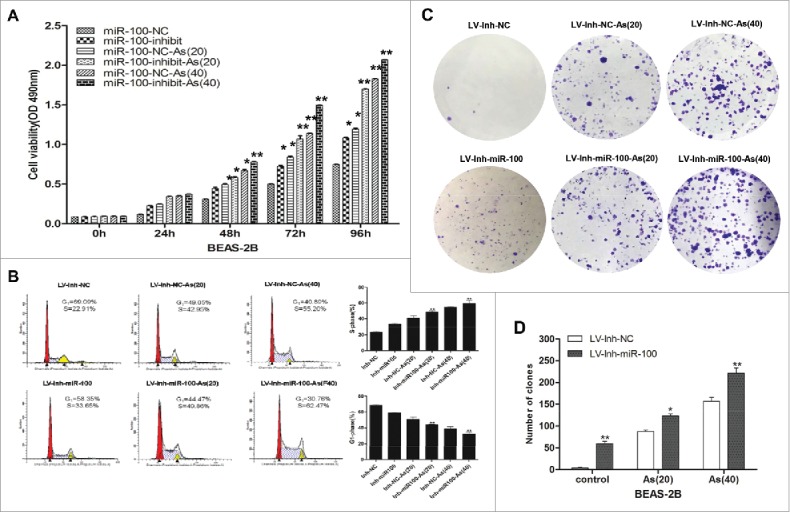
Inactivation of miR-100, combined with arsenic treatment, promotes proliferation of BEAS-2B cells. A. BEAS-2B cells with miR-100 inhibition were exposed to 0 or 0.25 μM As_2_O_3_ for 20, and 40 passages. MTT assay was performed to investigate the cell proliferation. The combination of miR-100 inhibition and chronic arsenic treatment promote the cell proliferation significantly. * p < 0.05, **p < 0.01. B. Cell cycle analysis by using flow cytometry system suggested that inhibition of miR-100 accelerated cell re-entering into the S phase, especially in as BEAS-2B(miR-100-inhibitor)-AS(40) cells.**p < 0.01. C. The colony-formation assay revealed that colony number and colony size were increased following inhibition of miR-100 in BEAS-2B cells, similar trend was observed in the AS-treated cells(AS (40) and AS (20)) when the expression of miR-100 was inhibited.*p < 0.05;**p < 0.01.

Increased proliferation is an important feature of cancer cells that is essential for the formation of primary tumors.^21^ In our study, we observed the effect on the cell proliferation of BEAS-2B after silencing the expression of miR-100 by using miR-100 inhibitor lentivirus expression vector (miR-100 -inhibitor). Cell proliferation was measured by MTT assay (Fig. 1A).We found that the proliferation ability of BEAS-2B cells increased after the inactivation of miR-100. It has been established in the literature that the proliferation of BEAS-2B cells increased when chronically exposed to sodium arsenite. To study whether arsenic can effect synergistically with miR-100 inhibited, the BEAS-2B cells transfected with miR-100 inhibitor lentivirus expression vector were incubated with or without 0.25 μM arsenic for 10 weeks (AS 20 passages) and 20 weeks (AS 40 passages). Compared with untreated group, the cell proliferation ability was significantly increased after low concentration of arsenic exposure (Fig. 1A and B).

**Figure 2.**
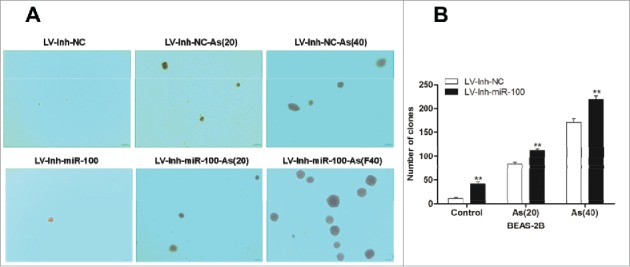
Inactivation of miR-100 combined with arsenic treatment promotes anchorage- independent growth of BEAS-2B cells. Left panel: The soft agar colony formation assay shows that inhibition of miR-100 promotes anchorage-independent growth of BEAS-2B cells. Right panel: Quantitative analysis of the agar colony formation. **p < 0.01.

Next, the effect of miR-100 inhibition on cell cycle progress of BEAS-2B cells was determined by flow cytometry (Fig. 1B). Compared with BEAS-2B/miR-NC cells, BEAS-2B/miR-100-inhibition cells showed the increased percentage of S phase cells and decreased percentage of G_0_/G_1_ phase cells. However, the percentage of G_2_/M phase cells showed no statistically difference between those 2 groups. To further determine the synergistic effect of chronic arsenic treatment combined with miR-100 inactivation, the cells were treated with arsenic for 20 passages and 40 passages, respectively. Similarly, we found that chronic arsenic treatment after miR-100 inactivation contributed the BEAS-2B cells to re-enter the cell cycle(S phase).

The effects of miR-100 inactivation on the colony-formation capacity of BEAS-2B cell was determined by colony-formation assay (Fig. 1C-D). The results showing that the colony number and colony size were increased following inhibition of miR-100 in BEAS-2B cells. Similar trend was observed in the chronic arsenic treated cells (AS [40] and AS [20]) after the miR-100 inactivation.

Taken together, these results confirmed that inhibition of miR-100 promoted proliferation of BEAS-2B cells, and chronic arsenic treatment increased BEAS-2B cell proliferation. The inactivation of miR-100 and chronic arsenic treatment might play synergistic role in this process.

### Inactivation of miR-100 combined with chronic arsenic treatment promotes anchorage- independent growth of BEAS-2B cells

Anchorage-independent growth is a characteristic gained by cancer cells.^22^ In this study, we observed that inhibition of miR-100 promoted anchorage-independent cell growth in soft agar (Fig. 2). The colony number and size was significantly increased after the inactivation of miR-100 in BEAS-2B cells. Furthermore, chronic arsenic treatment promotes the anchorage-independent growth of BEAS-2B cells after the miR-100 inactivation, indicated by the increased colony size and colony number.

### Inactivation of miR-100 combined with arsenic promotes migration of BEAS-2B cells

**Figure 3.**
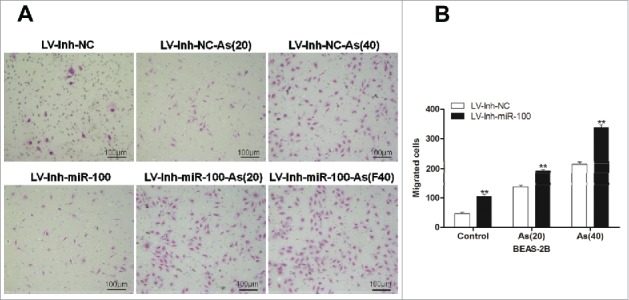
Inactivation of miR-100 combined with arsenic treatment promotes migration of BEAS-2B cells. Left panel: Transwell assays were performed to evaluate the migratory capabilities of the cells untransfected, transfected with either a miR-100–3p inhibitor (Inh-miR-100) or a miR-100–3p inhibitor control (Inh-NC) with or without chronic arsenic treatment. Similar trend was observed in the AS-treated cells when the expression of miR-100 was inhibited, especially in as BEAS-2B (Inh-miR-100)-AS (20) or AS (40) cells. Right panel: Quantitative analysis of the migration rates. **p < 0.01.

Cell invasion is a significant aspect of cancer progression.^14^ To investigate whether inhibition of miR-100 promoted migration of BEAS-2B cells, we evaluated BEAS-2B cell migration by using transwell assay. As showed in Fig. 3, inhibition of miR-100 promoted the migration of BEAS-2B cells by approximately 50.5% compared with control group. Moreover, numerous studies had documented that arsenic induced cell malignant transformation, i.e., promoted cell proliferation and migration at low concentration. To determine whether arsenic promoted migration of BEAS-2B cells after miR-100 inactivation, the cells were treated with low dose of arsenic chronically. As expected, the migration of BEAS-2B cells treated with arsenic after miR-100 inactivation was significantly increased when compared with control group. Moreover, the promoting effect was associated with the time of the As_2_O_3_ treatment.

These results demonstrated that inhibition of miR-100 promoted the ability of migration of BEAS-2B cells, while chronic arsenic treatment increased migration synergistically.

### Inactivation of miR-100 combined with chronic arsenic treatment promotes tumorigenesis of BEAS-2B cells in mice

**Figure 4.**
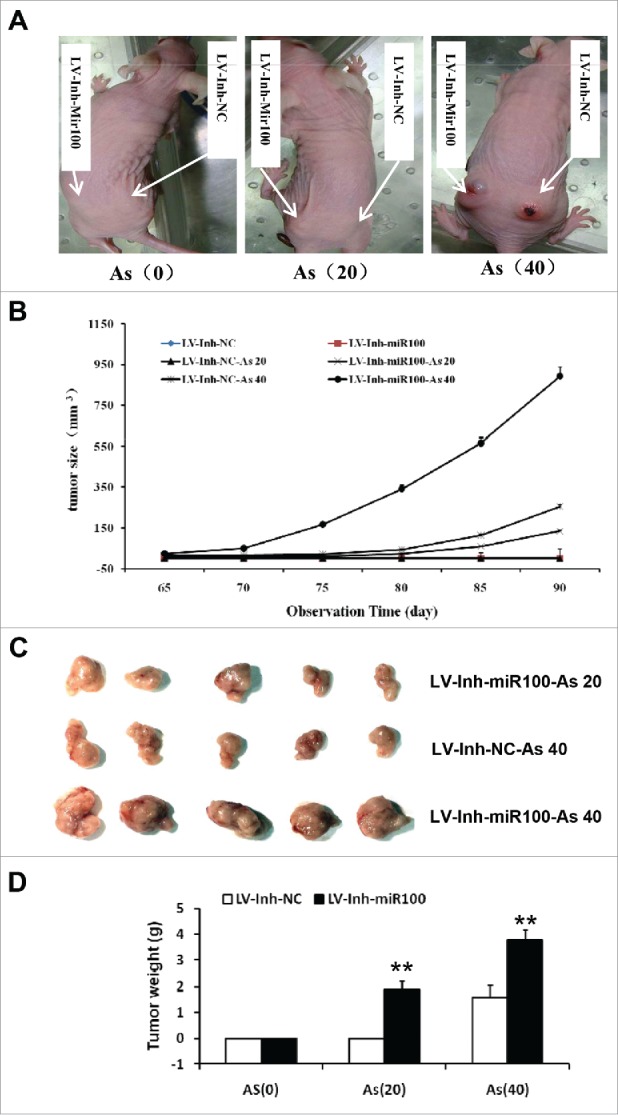
Inactivation of miR-100 combined with arsenic treatment promotes mammary tumor growth of BEAS-2B cells. A. the representative image showed mammary tumors from the miR-100 inactivation (Lv-Inh-miR100.) BEAS-2B cells with or without chronic arsenic treatment (0.25 μM) for 0, 20, 40 passages. B. Tumor size was monitored every 5 d after injection of BEAS-2B cells. C. Solid tumors were removed after the sacrifice at 90 d. The representative image showed mammary tumor size. D. Solid tumors from BEAS-2B cells were removed, and their weights were determined after sacrifice. The data were presented as the mean ± SEM (n = 5/group) *p < 0.05.

To further confirm the observed in vitro effects of the inactivation of miR-100 and the chronic arsenic treatment on the proliferation, viability, and invasiveness of the BEAS-2B cells, we investigated its roles in the growth of mammary tumors in vivo using a tumor xenograft model. The miR-100 inactivation BEAS-2B cells with or without chronic arsenic treatment were injected into BALB/C nude mice. Mammary tumors were detected after the tumor implantation (Fig. 4). The chronic arsenic treatment combined with miR-100 inactivation enhanced tumor growth. As shown in Fig. 4A, the tumor growth was promoted after the chronic arsenic treatment, and this effect was significantly enhanced by the inactivation of miR-100. The mammary tumors were removed after 90 d for further measurement. As illustrated in Fig. 4B-D, the miR-100 inactivation combined with chronic arsenic treatment significantly increased the weight of the mammary tumors.

### Inactivation of miR-100 combined with chronic arsenic treatment induces the EMT like transition

**Figure 5.**
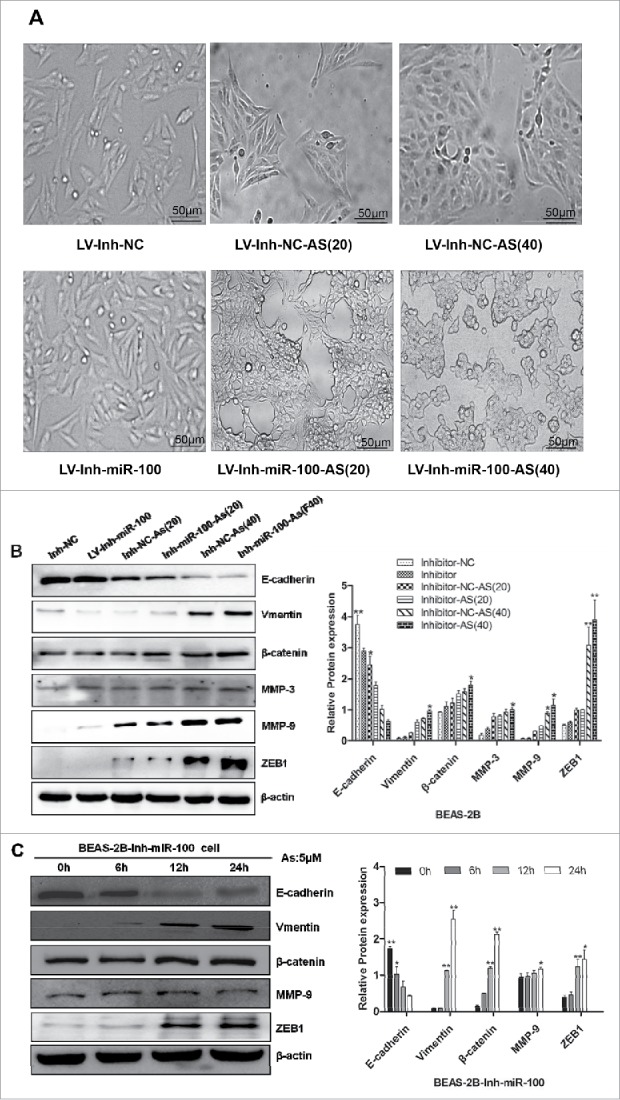
Morphological changes and Western Blotting evidences suggested that EMT is involved in the process. A. BEAS-2B cells with inhibited miR-100 were exposed to 0 or 0.25 μM of As_2_O_3_ for 0, 20, or 40 passages, and typical images with or without treatment are shown. Note the morphological shift of BEAS-2B cells from epithelial-like to mesenchymal-like when miR-100 inhibition. B, C. Chronic or acute exposure to As_2_O_3_ also induces the EMT-like phenotypical shift. Left panel: BEAS-2B cells with inhibited miR-100 were exposed to 0 or 0.25 μM of arsenic for 20, or 40 passages, or 5 μM of arsenic for 0, 6, 12, 24h. Western Blotting was performed to determine the protein expression levels of E-cadherin, Vimentin, MMP-3, MMP-9, β-catenin, and ZEB-1. Note that epithelial marker E-cadherin was inhibited by arsenic, whereas mesenchymal markers (Vimentin, MMP-3, and MMP-9) were upregulated. Right panel: Protein expression was quantified by band intensity and normalized to β-actin. *p < 0.05; **p < 0.01.

Literatures indicated that miR-100 is a novel EMT inducer, and validated in human tumors that miR-100 correlates with EMT-associated markers.^23^ It has been reported that chronic exposure to arsenic caused EMT during arsenic-induced malignant transformation.^24^ Consistently, in the process of chronic arsenic exposure, we observed that chronic arsenic treatment of BEAS-2B cells, when the expression of miR-100 was inhibited, underwent a marked morphologic change, i.e., from epithelial to spindle-like mesenchymal morphology (Fig. 5A).

To further confirm these observations, we next examined the expression of epithelial and mesenchymal markers by using western blotting. These results proved that treatment of BEAS-2B cells with arsenic, after inhibiting the expression of miR-100, resulted in EMT, as evidenced by reduction of the epithelial marker E-cadherin, and induction of the mesenchymal markers vimentin, ZEB1, and the matrix metalloproteinases MMP-3, MMP-9, and nuclear β-catenin.

To test whether these effects were due to the chronic exposure of arcsine, an acute As_2_O_3_ stimulating experiment was performed. BEAS-2B cells with miR-100 inactivation were exposed to 5 μM As_2_O_3_ in the short term, i.e., 0, 6, 12, and 24 h. We then examined the expression of EMT markers in treated cells. The results showed that the epithelial marker (E-cadherin) level was decreased, while mesenchymal markers (vimentin, ZEB1) and MMP-3, MMP-9, β-catenin levels were increased, similar results was observed in the BEAS-2B cells with chronic As_2_O_3_ exposure (Fig. 5C).

Overall, the morphological and molecular changes have all suggested that BEAS-2B cells with the inactivation of miR-100 underwent an EMT process after the As_2_O_3_ exposure.

## Discussion

Lung carcinogenesis is a long-term process, during which cells gain malignant biologic behavior, including increased cell proliferation, adhesion, migration, and invasion ability.^21^^,^^25^^,^^26^

Human beings are exposed to arsenite (AS) through environmental, medical, and occupational sources. Acute and chronic AS exposure via drinking water has been reported in many countries of the world. There is sufficient epidemiological evidence to support a causal association between AS exposure and human disease. Long-term exposure to AS through contaminated drinking water increases the risk of skin, lung, bladder, liver, and prostate cancers.^21^^,^^24^^,^^26^ Li Y reported that, a low concentration (1.0 μM) of AS induces anchorage- independent growth of HaCaT cells in soft agar.^21^ In our previous study, the HT-29 cell lines were exposed to 0 to 15 nM AS for 15 weeks and 30 weeks. The AS chronic exposure significantly promotes the cell viability, proliferation, migration, and adhesion in vitro and in vivo. In the present study, the effect of chronic AS exposure on carcinogenesis was investigated by using human bronchial epithelial cells, therefore, the higher concentration of 0.25 μM was chosen. In our study, we have established BEAS-2B cell lines with stable expression of miR-100 inhibitor, and then treated the cells with 0.25 μM arsenic for 10 weeks (AS 20 passages) and 20 weeks (AS 40 passages). We demonstrated that inhibition of miR-100 expression in BEAS-2B cells led to enhancement of cell proliferation and migration. Furthermore, to understand the synergistic effect of arsenic on BEAS-2B cells after inhibition of miR-100, the cells were treated with arsenic chronically. The results shown that chronic arsenic treatment promoted the malignant transformation of BEAS-2B cell after inhibition of miR-100 expression.

Arsenic exposure is a risk factor of human lung cancer. Exposure to inorganic arsenic was associated with the occurrence of lung cancer in a dose dependent manner in the residents with low methylation capacity in Taiwan.^27^ Mendez observed, that water arsenic concentrations were significant and positively associated with female lung cancer.^28^ Pratheeshkumar reported that, the arsenic induced lung cancer was induced by ROS-dependent activation of STAT3,^3^ while another study showed that arsenic bound to series of antioxidant proteins, such as peroxiredoxin, peroxide reductase, glutathione reductase, and glyceraldehyde-3-phosphate dehydrogenase.^30^ These observation revealed that the arsenic uptake by living cells increased the reactive oxidative stresses, and led to chromosome abnormalities and mutation,^31^ which might include the micro RNA expression.

The arsenic exposure affected on oncogenic transformation via affect the miR-100 expression. MiR-100 was downregulated in the lung cancer, and considered as a diagnostic bio-marker.^31^ The low miR-100 might be a poor prognostic factor in non-small cell lung cancer patients. Luo reported that, overexpression of miR-100 in non-small cell lung cancer cell inhibited the cancer growth, migration, and chemo-sensitivity through FGFR3.^32^ Xiao observed that the HOXA1 mediated the chemo-resistance of small cell lung cancer under the regulation of MiR-100.^29^ In the present study, the miR-100 inactivation promoted arsenic induced carcinogenesis in human lung bronchial epithelial cells, which might reveal the oncogenic transformation of lung disease under the arsenic exposure.

MiR-100 could induce EMT in human breast tumors.^23^ Chronic exposure to arsenic caused EMT during arsenic-induced malignant transformation.^24^ In our study, we observed that EMT is involved in inactivation of miR-100 and chronic arsenic treated BEAS-2B cells. EMT was considered to be a key early event in tumor invasion and metastasis,^33^ and was characterized by loss of epithelial cell apical-basal polarity, downregulation of epithelial markers including E-cadherin, and dissolution of cell-to-cell junctions. These changes promoted an adhesion switch to predominately cell-matrix interactions, were accompanied by drastic morphological changes, and were associated with the upregulation of a variety of cytoskeletal proteins that contribute to increased cell motility^34^^,^^35^ In our study, miR-100 inactivation in combination with chronic arsenic treatment of BEAS-2B caused EMT phenotype, a sign with more malignant features, particularly associated with increased metastatic potential.^36-38^

In this study, we identified as an EMT inducer, and demonstrated that inactivation of miR-100 in BEAS-2B cells led to significant enhancement of cell proliferation and migration. Our findings may help us to better understand the pathogenesis of lung cancer and facilitate the development of miRNA-directed diagnostics and cancer prevention.

## References

[1] Smith RA, Manassaram-Baptiste D, Brooks D, Doroshenk M, Fedewa S, Saslow D, Brawley OW, Wender R. Cancer screening in the United States, 2015: a review of current American cancer society guidelines and current issues in cancer screening. CA Cancer J Clin 2015; 65(1):30-54; PMID:25581023; doi: 10.3322/caac.21261. 25581023

[2] Arslan B, Djamgoz MB, Akun E ARSENIC: A review on exposure pathways, accumulation, mobility and transmission into the human food chain. Rev Environ Contam Toxicol 2017; 243:27-51; PMID:28005215; doi:10.1007/398_2016_18. 28005215

[3] Pratheeshkumar P, Son YO, Divya SP, Wang L, Zhang Z, Shi X Oncogenic transformation of human lung bronchial epithelial cells induced by arsenic involves ROS-dependent activation of STAT3-miR-21-PDCD4 mechanism. Sci Rep 2016; 6:37227; PMID:27876813; https://doi.org/10.1038/srep37227 27876813 PMC5120334

[4] Cogliano VJ, Baan R, Straif K, Grosse Y, Lauby-Secretan B, El Ghissassi F, Bouvard V, Benbrahim-Tallaa L, Guha N, Freeman C, et al. Preventable exposures associated with human cancers. J Natl Cancer Inst 2011; 103:1827-39; PMID:22158127; https://doi.org/10.1093/jnci/djr483 22158127 PMC3243677

[5] Cazaux C [Genetic instability as a driver for oncogenesis]. Bull Cancer 2010; 97:1241-51; PMID:21084240; https://doi.org/10.1684/bdc.2010.1202 21084240

[6] Herceg Z, Vaissiere T Epigenetic mechanisms and cancer: An interface between the environment and the genome. Epigenetics 2011; 6:804-19; PMID:21758002; https://doi.org/10.4161/epi.6.7.16262 21758002

[7] Alipoor SD, Adcock IM, Garssen J, Mortaz E, Varahram M, Mirsaeidi M, Velayati A The roles of miRNAs as potential biomarkers in lung diseases. Eur J Pharmacol 2016; 791:395-404; PMID:27634639; https://doi.org/10.1016/j.ejphar.2016.09.015 27634639 PMC7094636

[8] Qin C, Huang RY, Wang ZX Potential role of miR-100 in cancer diagnosis, prognosis, and therapy. Tumour Biol 2015; 36:1403-9; PMID:25740059; https://doi.org/10.1007/s13277-015-3267-8 25740059

[9] Leite KR, Tomiyama A, Reis ST, Sousa-Canavez JM, Sanudo A, Dall'Oglio MF, Camara-Lopes LH, Srougi M MicroRNA-100 expression is independently related to biochemical recurrence of prostate cancer. J Urol 2011; 185:1118-22; PMID:21255804; https://doi.org/10.1016/j.juro.2010.10.035 21255804

[10] Li BH, Zhou JS, Ye F, Cheng XD, Zhou CY, Lu WG, Xie X Reduced miR-100 expression in cervical cancer and precursors and its carcinogenic effect through targeting PLK1 protein. Eur J Cancer 2011; 47:2166-74; PMID:21636267; https://doi.org/10.1016/j.ejca.2011.04.037 21636267

[11] Liu J, Lu KH, Liu ZL, Sun M, De W, Wang ZX MicroRNA-100 is a potential molecular marker of non-small cell lung cancer and functions as a tumor suppressor by targeting polo-like kinase 1. BMC Cancer 2012; 12:519; PMID:23151088; https://doi.org/10.1186/1471-2407-12-519 23151088 PMC3521172

[12] Marshall G, Ferreccio C, Yuan Y, Bates MN, Steinmaus C, Selvin S, Liaw J, Smith AH Fifty-year study of lung and bladder cancer mortality in Chile related to arsenic in drinking water. J Natl Cancer Inst 2007; 99:920-8; PMID:17565158; https://doi.org/10.1093/jnci/djm004 17565158

[13] Wang S, Xue S, Dai Y, Yang J, Chen Z, Fang X, Zhou W, Wu W, Li Q Reduced expression of microRNA-100 confers unfavorable prognosis in patients with bladder cancer. Diagn Pathol 2012; 7:159; PMID:23173870; https://doi.org/10.1186/1746-1596-7-159 23173870 PMC3539897

[14] Thiery JP Epithelial-mesenchymal transitions in tumour progression. Nat Rev Cancer 2002; 2:442-54; PMID:12189386; https://doi.org/10.1038/nrc822 12189386

[15] Yang J, Weinberg RA Epithelial-mesenchymal transition: At the crossroads of development and tumor metastasis. Dev Cell 2008; 14:818-29; PMID:18539112; https://doi.org/10.1016/j.devcel.2008.05.009 18539112

[16] Park SM, Gaur AB, Lengyel E, Peter ME The miR-200 family determines the epithelial phenotype of cancer cells by targeting the E-cadherin repressors ZEB1 and ZEB2. Genes Dev 2008; 22:894-907; PMID:18381893; https://doi.org/10.1101/gad.1640608 18381893 PMC2279201

[17] Shimono Y, Zabala M, Cho RW, Lobo N, Dalerba P, Qian D, Diehn M, Liu H, Panula SP, Chiao E, et al. Downregulation of miRNA-200c links breast cancer stem cells with normal stem cells. Cell 2009; 138:592-603; PMID:19665978; https://doi.org/10.1016/j.cell.2009.07.011 19665978 PMC2731699

[18] Zhang J, Ma L MicroRNA control of epithelial-mesenchymal transition and metastasis. Cancer Metastasis Rev 2012; 31:653-62; PMID:22684369; https://doi.org/10.1007/s10555-012-9368-6 22684369 PMC3686549

[19] Ocana OH, Corcoles R, Fabra A, Moreno-Bueno G, Acloque H, Vega S, Barrallo-Gimeno A, Cano A, Nieto MA Metastatic colonization requires the repression of the epithelial-mesenchymal transition inducer Prrx1. Cancer Cell 2012; 22:709-24; PMID:23201163; https://doi.org/10.1016/j.ccr.2012.10.012 23201163

[20] Mani SA, Guo W, Liao MJ, Eaton EN, Ayyanan A, Zhou AY, Brooks M, Reinhard F, Zhang CC, Shipitsin M, et al. The epithelial-mesenchymal transition generates cells with properties of stem cells. Cell 2008; 133:704-15; PMID:18485877; https://doi.org/10.1016/j.cell.2008.03.027 18485877 PMC2728032

[21] Li Y, Xu Y, Ling M, Yang Y, Wang S, Li Z, Zhou J, Wang X, Liu Q mot-2-Mediated cross talk between nuclear factor-B and p53 is involved in arsenite-induced tumorigenesis of human embryo lung fibroblast cells. Environ Health Perspect 2010; 118:936-42; PMID:20199942; https://doi.org/10.1289/ehp.0901677 20199942 PMC2920912

[22] Chang Q, Pan J, Wang X, Zhang Z, Chen F, Shi X Reduced reactive oxygen species-generating capacity contributes to the enhanced cell growth of arsenic-transformed epithelial cells. Cancer Res 2010; 70:5127-35; PMID:20516118; https://doi.org/10.1158/0008-5472.CAN-10-0007 20516118 PMC4048957

[23] Chen D, Sun Y, Yuan Y, Han Z, Zhang P, Zhang J, You MJ, Teruya-Feldstein J, Wang M, Gupta S, et al. miR-100 induces epithelial-mesenchymal transition but suppresses tumorigenesis, migration and invasion. PLoS Genet 2014; 10:e1004177; PMID:24586203; https://doi.org/10.1371/journal.pgen.1004177 24586203 PMC3937226

[24] Sun JL, Chen DL, Hu ZQ, Xu YZ, Fang HS, Wang XY, Kan L, Wang SY Arsenite promotes intestinal tumor cell proliferation and invasion by stimulating epithelial-to-mesenchymal transition. Cancer Biol Ther 2014; 15:1312-9; PMID:25010681; https://doi.org/10.4161/cbt.29685 25010681 PMC4130724

[25] Park WH MAPK inhibitors and siRNAs differentially affect cell death and ROS levels in arsenic trioxide-treated human pulmonary fibroblast cells. Oncol Rep 2012; 27:1611-8; PMID:22293863; https://doi.org/10.3892/or.2012.1661 22293863

[26] Chang Q, Chen B, Thakur C, Lu Y, Chen F Arsenic-induced sub-lethal stress reprograms human bronchial epithelial cells to CD61 cancer stem cells. Oncotarget 2014; 5:1290-303; PMID:24675390; https://doi.org/10.18632/oncotarget.1789 24675390 PMC4012730

[27] Hsu KH, Tsui KH, Hsu LI, Chiou HY, Chen CJ Dose-response relationship between inorganic arsenic exposure and lung cancer among arseniasis residents with low methylation capacity. Cancer Epidemiol Biomarkers Prev 2017; 26(5):756-761; PMID:28007985; http://doi: 10.1158/1055-9965.EPI-16-0281. 28007985

[28] Mendez WM Jr, Eftim S, Cohen J, Warren I, Cowden J, Lee JS, Sams R Relationships between arsenic concentrations in drinking water and lung and bladder cancer incidence in U.S. counties. J Expo Sci Environ Epidemiol 2017; 27(3):235-243; PMID:27901016; doi: 10.1038/jes.2016.58. 27901016

[29] Xiao F, Bai Y, Chen Z, Li Y, Luo L, Huang J, Yang J, Liao H, Guo L Downregulation of HOXA1 gene affects small cell lung cancer cell survival and chemoresistance under the regulation of miR-100. Eur J Cancer 2014; 50:1541-54; PMID:24559685; https://doi.org/10.1016/j.ejca.2014.01.024 24559685

[30] Yan X, Li J, Liu Q, Peng H, Popowich A, Wang Z, Li XF, Le XC p-Azidophenylarsenoxide: An arsenical “bait” for the in situ capture and identification of cellular arsenic-binding proteins. Angew Chem Int Ed Engl 2016; 55:14051-6; PMID:27723242; https://doi.org/10.1002/anie.201608006 27723242

[31] Lee CH, Yu HS Role of mitochondria, ROS, and DNA damage in arsenic induced carcinogenesis. Front Biosci (Schol Ed) 2016; 8:312-20; PMID:27100709; https://doi.org/10.2741/s465 27100709

[32] Luo J, Chen B, Ji XX, Zhou SW, Zheng D Overexpression of miR-100 inhibits cancer growth, migration, and chemosensitivity in human NSCLC cells through fibroblast growth factor receptor 3. Tumour Biol 2016; 37(12):15517-24; PMID:26314855; https://doi.org/10.1007/s13277-015-3850-z 26314855

[33] Thiery JP, Acloque H, Huang RY, Nieto MA Epithelial-mesenchymal transitions in development and disease. Cell 2009; 139:871-90; PMID:19945376; https://doi.org/10.1016/j.cell.2009.11.007 19945376

[34] Lee JM, Dedhar S, Kalluri R, Thompson EW The epithelial-mesenchymal transition: New insights in signaling, development, and disease. J Cell Biol 2006; 172:973-81; PMID:16567498; https://doi.org/10.1083/jcb.200601018 16567498 PMC2063755

[35] Eckert MA, Lwin TM, Chang AT, Kim J, Danis E, Ohno-Machado L, Yang J Twist1-induced invadopodia formation promotes tumor metastasis. Cancer Cell 2011; 19:372-86; PMID:21397860; https://doi.org/10.1016/j.ccr.2011.01.036 21397860 PMC3072410

[36] Fidler IJ The pathogenesis of cancer metastasis: The ‘seed and soil’ hypothesis revisited. Nat Rev Cancer 2003; 3:453-8; PMID:12778135; https://doi.org/10.1038/nrc1098 12778135

[37] Cooke VG, LeBleu VS, Keskin D, Khan Z, O'Connell JT, Teng Y, Duncan MB, Xie L, Maeda G, Vong S, et al. Pericyte depletion results in hypoxia-associated epithelial-to-mesenchymal transition and metastasis mediated by met signaling pathway. Cancer Cell 2012; 21:66-81; PMID:22264789; https://doi.org/10.1016/j.ccr.2011.11.024 22264789 PMC3999522

[38] Wang L, Wu Y, Lin L, Liu P, Huang H, Liao W, Zheng D, Zuo Q, Sun L, Huang N, et al. Metastasis-associated in colon cancer-1 upregulation predicts a poor prognosis of gastric cancer, and promotes tumor cell proliferation and invasion. Int J Cancer 2013; 133:1419-30; PMID:23457029; https://doi.org/10.1002/ijc.28140 23457029

